# A rare case of asymptomatic right coronary artery ectasia associated with giant aneurysm

**DOI:** 10.1093/omcr/omac023

**Published:** 2022-03-16

**Authors:** Takanori Hishikawa, Takeki Ohashi, Masao Tadakoshi, Yuji Kamikawa, Soichiro Kageyama, Akinori Kojima, Kaoru Hioki, Hirotaka Yamauchi

**Affiliations:** Cardiovascular surgery, Nagoya Tokushukai General Hospital, Aichi, Japan; Cardiovascular surgery, Nagoya Tokushukai General Hospital, Aichi, Japan; Cardiovascular surgery, Nagoya Tokushukai General Hospital, Aichi, Japan; Cardiovascular surgery, Nagoya Tokushukai General Hospital, Aichi, Japan; Cardiovascular surgery, Nagoya Tokushukai General Hospital, Aichi, Japan; Cardiovascular surgery, Nagoya Tokushukai General Hospital, Aichi, Japan; Cardiovascular surgery, Nagoya Tokushukai General Hospital, Aichi, Japan; Cardiovascular surgery, Nagoya Tokushukai General Hospital, Aichi, Japan

## Abstract

A rare case of giant coronary artery ectasia associated with coronary artery aneurysm was recognized. A 69-year-old woman presented with an ischemic electrocardiogram changes during a medical check-up. Coronary computed tomography angiography showed right coronary artery (RCA) ectasia associated with a giant aneurysm originating from the distal RCA. She was asymptomatic and exhibited no risk factors, including Kawasaki disease, hypertension, diabetes mellitus or family history. The patient underwent surgery for giant coronary aneurysms to prevent rupture. The aneurysm was on the peripheral side of the right coronary artery; hence, coronary artery bypass was not performed. The patient’s postoperative course was uneventful. Histopathological examination of the aneurysm revealed degeneration due to atherosclerosis. She was prescribed warfarin and aspirin for thrombus prevention.

## INTRODUCTION

Coronary artery ectasia (CAE) and coronary artery aneurysm (CAA) are aneurysmal dilatations of the coronary artery. Both of these diseases show dilatation of at least 1.5 times the diameter of normal coronary arteries [1, 2]. CAE is defined as diffuse dilatation of coronary arteries more than one-third of their length, and CAA is defined as dilatation of less than one-third of the length of the coronary artery. Although considered an infrequent finding during coronary angiography, the prevalence of CAE is reported to be between 0.3% and 5.3%; likewise, the prevalence of CAA is reported to be between 1.5% and 4.9% [2, 3]. Although the most common aetiology for the development of CAE and CAA has been found to be atherosclerosis, there have been few reports of CAE cases associated with CAA. We report a rare case of CAE and a large CAA, which is unparalleled among the reported cases.

## CASE REPORT

**Figure 1:**
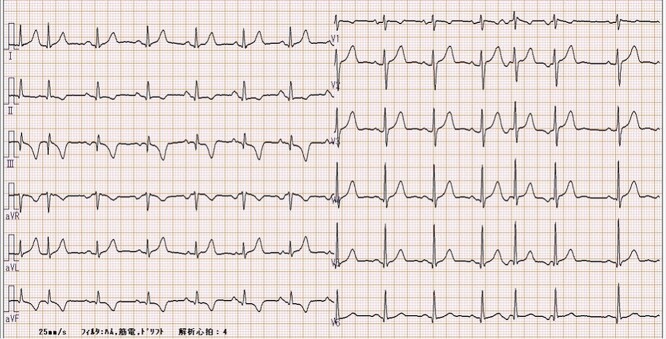
The electrocardiogram at the time of admission shows negative T waves in 2, 3 and aVf inductions.

**Figure 2:**
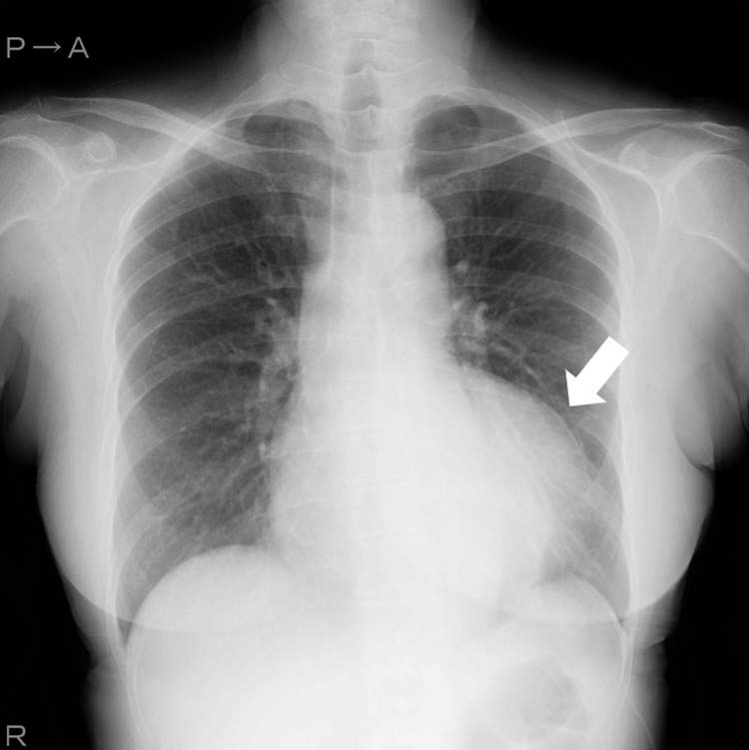
Chest radiography shows aneurysm as protruding mass.

**Figure 3:**
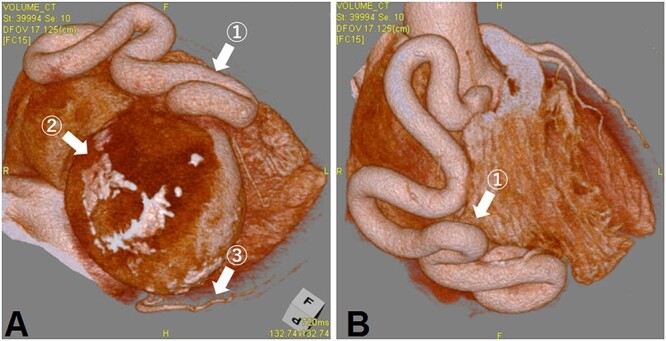
(A) The CAE is seen (①). The right coronary artery flows into the CAA with a diameter of 70 mm (②). A normal diameter left circumflex artery is also seen (③). (B) The CAE is seen (①).

**Figure 4:**
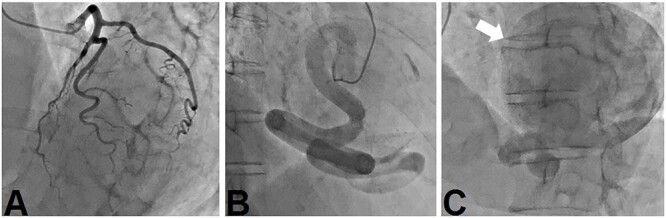
(A) There is no stenosis or dilation in the left coronary artery. (B) The right coronary artery is dilated and (C) flows into a large coronary aneurysm.

**Figure 5:**
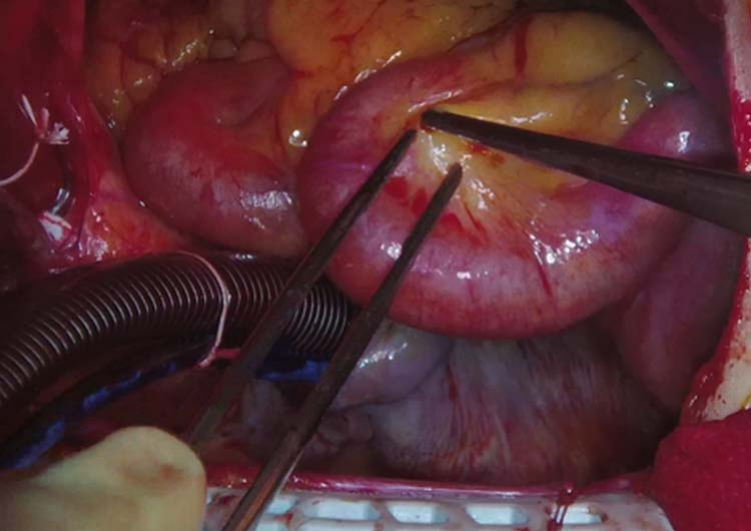
The ectatic right coronary artery is seen.

**Figure 6:**
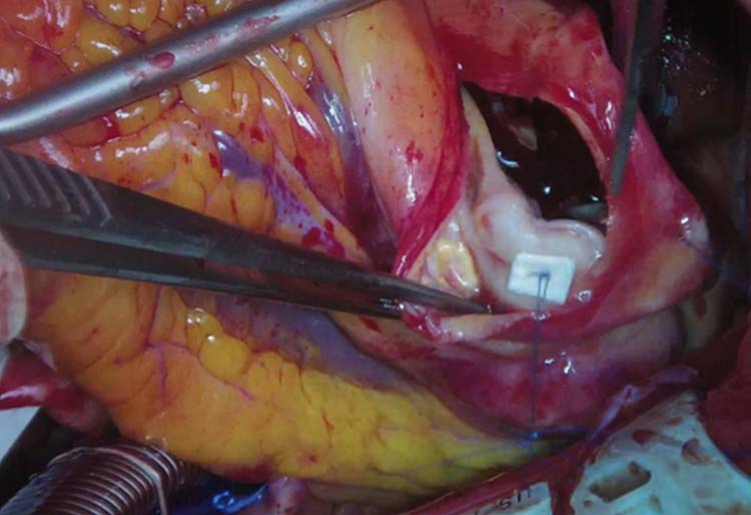
The aneurysm wall of a coronary aneurysm was incised. The white arrow indicates the inlet to the coronary aneurysm. The inlet was closed with 4-0 monofilament polypropylene.

**Figure 7:**
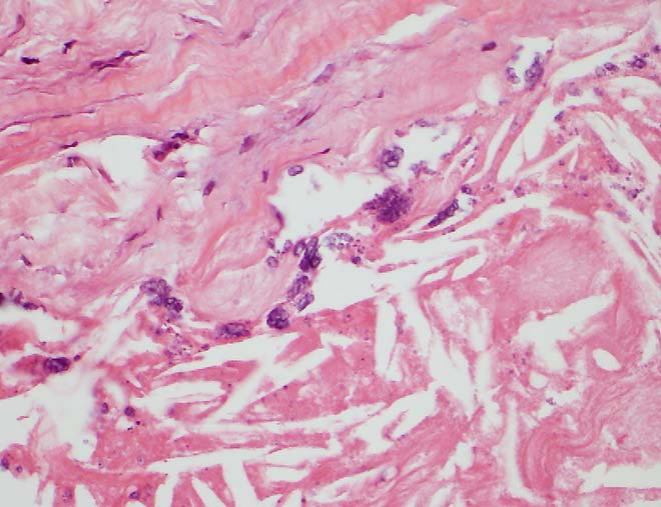
Histology of the right coronary artery aneurysm. Tunica intima expanded by atherosclerosis and tunica media densely infiltrated by inflammatory cells.

**Figure 8:**
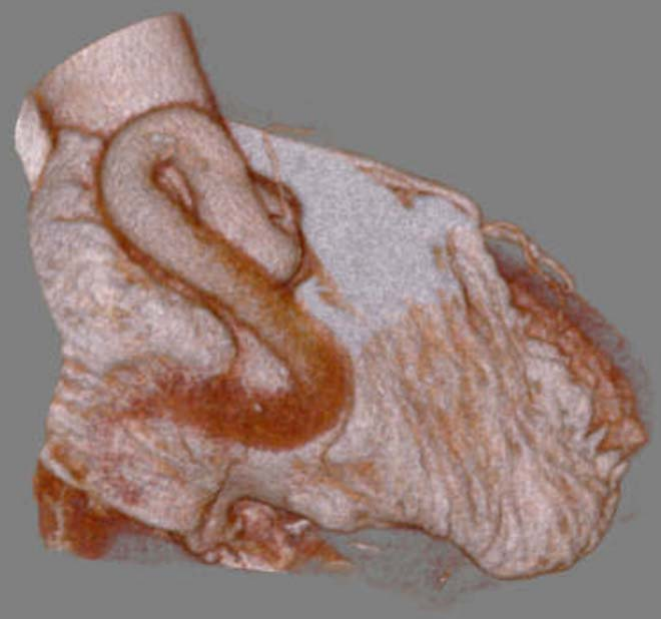
Postoperative coronary computed tomography shows no blood flow into the coronary aneurysm.

A 69-year-old woman presented with ischemic changes in the inferior lead (Fig. 1), as noted on electrocardiography during a medical check-up. The patient underwent an echocardiogram to evaluate ischaemia and was found to have a coronary aneurysm, and chest radiography showed a protruding coronary aneurysm (Fig. 2). Coronary computed tomography angiography showed right coronary artery (RCA) ectasia associated with a giant aneurysm originating from the distal RCA (Fig. 3A and B). The diameters of the ectasia and aneurysm were 15 and 70 mm, respectively. Coronary angiography showed no stenosis or dilatation in the left coronary artery (Fig. 4A), whereas the right CAE was observed along its entire length (Fig. 4B). A giant CAA was also observed (Fig. 4C). She was asymptomatic and exhibited no risk factors, including Kawasaki disease, hypertension, diabetes mellitus or family history. CAA was so large that an aneurysmectomy was performed to prevent rupture. The aneurysm and CAE (Fig. 5) were exposed to cardioplegic arrest and cardiopulmonary bypass performed through a median sternotomy. The aneurysmal wall was incised, and the inflow hole was confirmed with antegrade cardioplegia. There was neither an outflow nor fistula in the aneurysm. The inflow hole was closed with a pledgeted 4-0 polypropylene mattress suture inside the aneurysm without closing the coronary artery distal to the RCA (Fig. 6). The aneurysmal sac was then punctured. The patient’s postoperative course was uneventful. Histopathological examination of the aneurysm revealed degeneration due to atherosclerosis (Fig. 7). A coronary CT scan showed no inflow into the CAA postoperatively (Fig. 8). The patient was prescribed warfarin and aspirin for the prevention of thrombus in the dilated coronary arteries in the CAE to prevent myocardial infarction. The patient is still recovering without any postoperative complications and is doing well as an outpatient.

## DISCUSSION

A CAE or CAA can be asymptomatic and incidentally detected during a medical check-up.

However, a giant aneurysm can be symptomatic and can lead to life-threatening complications. Therefore, early diagnosis and treatment are necessary to save patients. However, until now, there has been no consensus on the optimal strategy for the treatment of CAE and CAA due to a lack of randomized, controlled clinical trials or official guidelines. The optimal strategy should be individualized, based on the size and morphology of the aneurysms and associated lesions, for each patient. In this case, since this patient did not have any symptoms, coronary ectasia and a huge aneurysm were incidentally found with electrocardiogram during a medical check-up. Because the size of the aneurysm was enormous and the risk of rupture was high, we performed surgical resection of the aneurysm. As the aneurysm was located in the peripheral RCA, we did not perform coronary artery bypass grafting. Atherosclerosis is considered the common etiologic factor responsible for CAE in adults, whereas Kawasaki disease is the most common cause in children and young adults [4]. In this case, the patient did not have a history of Kawasaki disease, and the histopathological results of the ectasia and aneurysms showed degeneration due to atherosclerosis. However, there were no risk factors for atherosclerosis or atherosclerotic lesions in other coronary arteries. There have also been reports of coronary aneurysms that developed after the primary coronary artery dissection [5]. Although CAA usually develops with sudden chest pain, such as acute coronary syndrome, and has a high fatality rate, it is possible that in our patient, this disease occurred asymptomatically, and the tunica media subsequently became vulnerable and formed a coronary aneurysm.

CAE is still present in this case. The presence of CAE predicts future cardiac events in patients with acute myocardial infarction. Doi *et al.* suggested that acute myocardial infarction patients with CAE are a high-risk subset who might benefit from a pharmacological approach to control the coagulation cascade [6]. Concomitant use of antiplatelet and anticoagulant medications should be used with caution because they increase the risk of bleeding events, but they may be effective in preventing myocardial infarction.

## CONFLICT OF INTEREST STATEMENT

None declared.

## ETHICAL APPROVAL

The studies have received an approval from our institutional ethics review board.

## CONSENT

All study participants provided informed consent.

## GUARANTOR

Takanori Hishikawa.
